# Microglia-Mediated Phagocytosis in Alzheimer’s Disease: Mechanisms, Heterogeneity, and Therapeutic Insights

**DOI:** 10.3390/biom15111629

**Published:** 2025-11-20

**Authors:** Halimatu Hassan, Charlotte Rawlinson, Yu-Long Lan, Stuart Jenkins, Ruoli Chen

**Affiliations:** 1 School of Allied Health Professions and Pharmacy, Keele University, Staffordshire ST5 5BG, UK; h.hassan@keele.ac.uk; 2 The ME Association, Gawcott, Buckinghamshire MK18 4DF, UK; rawlinson.c.t.b@gmail.com; 3 Department of Neurosurgery, Second Affiliated Hospital, School of Medicine, Zhejiang University, Hangzhou 310009, China; lanyulong@zju.edu.cn; 4 Neural Tissue Engineering Keele (NTEK), School of Medicine, Keele University, Staffordshire ST5 5BG, UK; s.i.jenkins@keele.ac.uk

**Keywords:** Alzheimer’s disease, microglia, phagocytosis, neuroinflammation, neuroprotection, neurodegeneration

## Abstract

Microglia are the resident immune cells of the CNS, maintaining brain homeostasis partially through phagocytosis. In Alzheimer’s disease (AD), microglial phagocytosis is significantly impaired, contributing to the accumulation of pathological aggregates. Microglial phenotypes are dynamic and can shift depending on the disease stage and local environment. While some subpopulations retain or enhance phagocytic activity, especially under inflammatory conditions, others lose their capacity to clear toxic debris effectively. This variability underscores the need for a more nuanced understanding of microglial regulation and function. This paper explores the dual role of microglial phagocytosis in AD and discusses the emerging insights into microglial heterogeneity and how phenotypic shifts affect phagocytic capacity throughout disease progression. A comprehensive understanding of microglial phagocytosis and its dysregulation in AD is essential for designing targeted treatments. Modulating microglial activity to enhance their protective roles without triggering harmful inflammation represents a promising direction for therapeutic intervention in AD.

## 1. Introduction

Microglia, the innate immune cells of the central nervous system (CNS), play essential roles in maintaining brain homeostasis, responding to injury, and defending against pathogens. Through specialized receptors, microglia continuously monitor microenvironment in the CNS, phagocytosing misfolded proteins, cellular debris, and apoptotic cells [1,2,3]. They also communicate with neurons, supporting neuronal function and synaptic remodeling [4]. Once considered a homogenous population, microglia are now recognized as highly heterogeneous, exhibiting diverse states shaped by both intrinsic factors (e.g., species, sex, genetic background) and extrinsic influences (e.g., pathogens, microbiota) [5].

In response to perturbations in the microenvironment, microglia rapidly migrate toward the triggering stimuli and become activated. Microglial activation refers to the morphological and functional transformation of microglia from a surveillant ramified state to a reactive amoeboid form, characterized by an enlarged cell body, retracted processes, and cytoplasmic vacuoles [6]. This morphological shift is associated with functional changes, including migration, antigen presentation, and enhanced phagocytic activity [7].

Efforts to more precisely characterize microglial activation are evolving. Recent advances in single-cell RNA sequencing (scRNA-seq) and mass cytometry technologies, such as Cytometry by Time-of-Flight (CyTOF), have facilitated the identification of numerous microglial states across developmental, aging, and disease contexts [8,9]. Notably, disease-associated microglia (DAMs)—a distinct response state—have been identified in both mouse models and human Alzheimer’s disease (AD) brains. DAMs are found clustered around amyloid plaques and are believed to contribute to the clearance of β-amyloid (Aβ), a key pathological feature of AD [10,11]. Importantly, scRNA-seq studies have demonstrated that microglial activation does not conform to the traditional M1/M2 dichotomy of pro- versus anti-inflammatory states [12,13]. Instead, microglia exhibit a high degree of plasticity in response to diverse pathological conditions, underscoring the complexity of their roles in neurodegenerative diseases such as AD. Gerrits et al. have identified three microglial subtypes in AD patients: homeostatic microglia, AD1-microglia, and AD2-microglia [12]. AD1-microglia, localized near Aβ plaques, exhibit features of DAM and are involved in functions such as phagocytosis and cell migration. In contrast, AD2-microglia may play neurotrophic roles [12].

AD is the most prevalent neurodegenerative disorder, accounting for approximately 60% of dementia cases worldwide [14]. With the aging global population, AD prevalence is projected to quadruple by 2050, potentially affecting over 100 million individuals [15]. Hallmark neuropathological features of AD include extracellular amyloid plaques, intracellular tau neurofibrillary tangles, microgliosis, astrogliosis, and progressive cerebral atrophy [16]. The widely accepted amyloid hypothesis posits that misfolded Aβ initiates a cascade of pathological processes, including tau aggregation, culminating in neurodegeneration [17].

Microglia are thought to exert both neuroprotective and neurotoxic effects in AD. Early in disease progression, they may facilitate the clearance of Aβ and cellular debris; however, chronic activation can lead to sustained neuroinflammation and collateral damage to healthy brain tissue [18]. Moreover, microglial phagocytic function appears to be impaired in AD, resulting in the accumulation of toxic waste and further microglial activation. Conversely, excessive or inappropriate synaptic pruning by microglia may contribute to cognitive decline [19].

A deeper understanding of microglial heterogeneity and their functional states in AD is essential for the development of targeted therapeutics. Modulating microglial pathways involved in phagocytosis presents a promising avenue for novel treatment strategies in AD and other neurodegenerative disorders.

In this review, we not only explore the multifaceted roles of microglia and their phagocytic activity in AD, but also examine how altered microglial phagocytosis contributes to disease progression and discuss emerging microglia-targeted therapeutic strategies.

## 2. Microglial Function and Phagocytosis

Microglia are responsible for a broad array of functions, including immune surveillance, synaptic pruning, and the clearance of apoptotic cells and cellular debris [20]. Two-photon imaging has revealed that even in their so-called “resting” state, microglia are highly dynamic and motile. They maintain a branched morphology that enables continuous surveillance of the surrounding microenvironment, leading to frequent and sustained contact with synapses [21].

One of the fundamental roles of microglia is phagocytosis—the engulfment and degradation of potentially harmful substances to prevent their accumulation and neurotoxicity. Phagocytosis, a process shared by other immune cells such as neutrophils, macrophages, dendritic cells, and B lymphocytes, involves the recognition, internalization, and lysosomal digestion of foreign material, apoptotic cells, or cellular debris [22]. While phagocytosis is traditionally viewed as a component of the innate immune response, it also intersects with adaptive immunity through microglial antigen presentation capabilities [22].

In the CNS, phagocytosis is predominantly carried out by microglia, though infiltrating monocytes can differentiate into microglia-like cells and contribute under certain pathological conditions [23]. Despite phenotypic and functional similarities to other tissue-resident macrophages, microglia are distinct in their developmental origin and in their adaptation to the unique CNS environment [24].

During early brain development, the generation of neurons and synapses occur at a rate that exceeds the final structural requirements. Microglia contribute to brain maturation by eliminating superfluous synapses in a process known as synaptic pruning [25]. This process is regulated by several signaling pathways. For instance, complement proteins C1q and C3 are deposited on immature or inactive synapses, tagging them for elimination [26]. CX3CL1 (Fractalkine), a chemokine expressed by neurons, signals through its receptor CX3CR1, which is predominantly expressed in microglia and regulates migration, adhesion, and survival [27]. Knockout studies of CX3CR1 in mice have shown increased dendritic spine density and deficits in hippocampal synaptic function, as well as impairments in social behavior and connectivity in adulthood [28].

Cytokines such as tumor necrosis factor-alpha (TNF-α) also play important roles in modulating synaptic strength and integrity [29]. Furthermore, microglial secretion of brain-derived neurotrophic factor (BDNF) contributes to synaptic plasticity by promoting phosphorylation of the neuronal tropomyosin receptor kinase B (TrkB) receptor, especially during critical periods of brain development [30].

In the adult brain, under non-infectious conditions, the primary phagocytic function of microglia is the clearance of dead cells. However, they are also capable of engulfing live, but stressed or damaged, neurons and precursors—a process termed “phagoptosis”. This mechanism contributes to the regulation of neuronal populations, synaptic remodeling, and maintenance of CNS homeostasis throughout development and into adulthood [25].

A specialized form of phagocytosis known as efferocytosis refers to the silent clearance of apoptotic cells. Unlike pathogen-induced phagocytosis, which often triggers inflammatory responses, efferocytosis promotes tissue repair and anti-inflammatory signaling. Notably, genetic disruption of microglial phagocytosis in the developing hippocampus resulted in fewer caspase-3-positive neurons, indicating that phagocytosis may also influence apoptosis itself [31].

**Figure 1:**
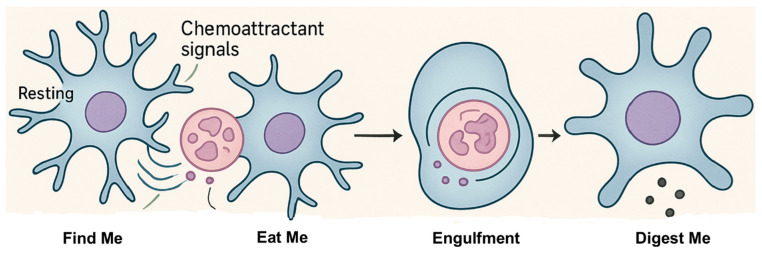
Phases of efferocytosis by microglia. Microglia clear apoptotic cells through four sequential phases: Find me—resting microglia detect “find-me” chemoattractants released by apoptotic cells; Eat me—microglia recognize “eat-me” signals such as phosphatidylserine on the apoptotic cell surface, initiating tethering; Engulfment—the apoptotic cell is internalized into a phagosome; Digest me—fusion of the phagosome with lysosomes forms a phagolysosome, where lysosomal hydrolases degrade cellular contents. Efferocytosis drives microglial activation, accompanied by characteristic morphological changes including process retraction and enlarged microglial cell bodies.

Efferocytosis occurs in three distinct stages. The first is the “find-me” phase, during which apoptotic cells release chemoattractant signals that recruit microglia. This is followed by the “eat-me” phase, where surface molecules such as phosphatidylserine mediate recognition and tethering between the microglia and apoptotic cell. The final stage, the “digest-me” phase, involves the enzymatic degradation of engulfed content within phagolysosomes [32] (Figure 1).

### 2.1. ”Find-Me” Phase

The first stage of efferocytosis involves the recruitment of phagocytic cells to apoptotic or damaged cells (Figure 1). This process is mediated by several molecular signals and receptors that guide microglia toward dying cells.

Triggering Receptor Expressed on Myeloid Cells 2 (TREM2) plays a crucial role in the non-inflammatory clearance of apoptotic neurons and is essential for microglial chemotaxis in response to neuronal injury [33,34,35,36]. TREM2 is specifically expressed by brain microglia and supports their survival, activation, and phagocytic capacity [34].

Purinergic receptor signaling is also implicated in microglial recruitment. Nucleotides such as UDP and ATP, released into the extracellular space by apoptotic neurons, act as “find-me” signals [37,38,39]. The P2Y6 receptor, a metabotropic receptor that binds UDP, is expressed constitutively on microglia and becomes upregulated following neuronal injury. Activation of P2Y6 promotes microglial chemotaxis and phagocytosis of apoptotic neurons [40]. Interestingly, microglia surrounding Aβ plaques in AD models show impaired P2Y6 signaling and reduced phagocytic function, suggesting a role for UDP-P2Y6 signaling in Aβ clearance [40].

CX3CL1 (Fractalkine), a chemokine released from neurons, also mediates microglial chemotaxis and synaptic pruning. Soluble CX3CL1, generated by ADAM10 cleavage, promotes the release of milk fat globule-EGF factor 8 (MFG-E8), which bridges apoptotic cells and phagocytes. MFG-E8 binds to phosphatidylserine on apoptotic cells via its C2 domain and to vitronectin receptors (αvβ3/5 integrins) on microglia through its Arginine–Glycine–Aspartic acid motif, facilitating engulfment [41,42,43,44].

The receptor tyrosine kinases Mer and Axl, part of the TAM (Tyro3, Axl, Mer) family, are also essential in this phase. Mice lacking Mer and Axl accumulate apoptotic neurons in neurogenic regions due to impaired microglial motility and recruitment [45].

### 2.2. “Eat-Me” Phase

#### 2.2.1. “Eat-Me” Signals

“Eat-me” signals are surface markers on apoptotic or stressed cells that trigger recognition and engulfment by phagocytes (Figure 1). They are critical for maintaining tissue homeostasis and preventing secondary necrosis and inflammation [46].

The best-characterized “eat-me” signal is phosphatidylserine (PS), typically confined to the inner leaflet of the plasma membrane. During apoptosis, scramblases redistribute PS to the outer leaflet, where it is recognized by phagocytic receptors such as TIM-4 and BAI1. TIM-4 stabilizes the phagosome post-engulfment, while BAI1 promotes phagosome formation [47,48].

Complement proteins, especially C1q, bind apoptotic cells and facilitate their recognition by complement receptors on microglia, triggering phagocytosis [49].

Neuron-specific enolase (NSE), released upon neuronal injury, and high mobility group box 1 (HMGB1), a nuclear damage-associated molecular patterns (DAMPs) molecule, can also act as “eat-me” signals. HMGB1 binds to the receptor for AGEs (RAGE) and Toll-like receptor (TLR)—4 on microglia to initiate phagocytosis. However, chronic HMGB1 signaling may drive neuroinflammation and exacerbate neurodegenerative processes [50,51].

#### 2.2.2. ”Do-Not-Eat-Me” Signals

In contrast to “eat-me” cues, “do-not-eat-me” signals protect healthy cells from unintended phagocytosis. One such molecule is CD47, which interacts with signal regulatory protein alpha (SIRPα) on microglia. This interaction activates SHP-1 (Src homology region 2 domain-containing phosphatase-1), a tyrosine phosphatase that inhibits phagocytosis [52,53]. This mechanism ensures selective clearance but may hinder debris removal in pathological states. For example, SIRPα-mediated inhibition impaired clearance of degenerating myelin in a model of experimental autoimmune encephalomyelitis (EAE), delaying axonal repair [54]. Another inhibitory cue is sialic acid on neuronal glycoproteins. These bind to Siglecs (sialic acid-binding immunoglobulin-like lectins) on microglia, suppressing phagocytosis and preventing excessive pruning during development [55].

### 2.3. ”Digest-Me” Phase

Following engulfment, the phagocytic process enters the “digest-me” phase, where the phagosome matures and fuses with lysosomes (Figure 1). This results in the enzymatic degradation and recycling of internalized material. While the early stages of efferocytosis are well characterized, the molecular mechanisms underlying phagosome maturation remain less understood. Studies in *Drosophila melanogaster* identified Draper, the homolog of *C. elegans* Ced-1 and human CD91/LRP1, as essential for phagosome maturation. Flies deficient in Draper accumulated apoptotic neurons and showed age-dependent neurodegeneration, which could be rescued by TORC1 (Target of Rapamycin Complex 1) activation or Atg1 (Autophagy-related gene 1) inhibition [56]. Importantly, Draper was not involved in dead cell recognition but specifically regulated the maturation stage. Notably, lysosomal degradation capacity declines with aging and during neurodegenerative diseases, impairing phagosome processing and contributing to cellular dysfunction [57].

## 3. Roles and Responses of Microglia to Alzheimer’s Disease Pathogenesis

Microglia play a crucial role in responding to AD pathogenesis. Reactive microglia are often found clustered near Aβ plaques in both AD mouse models [58] and human post-mortem brain tissues [59,60]. In vivo imaging studies further confirm that microglial activation correlates with Aβ and tau accumulation in AD brains [61]. Early in the disease, microglial activation likely exerts protective effects by migrating toward and engulfing Aβ and tau deposits [18]. However, prolonged exposure to these pathological proteins can convert microglia from a protective to a dysfunctional state, thereby contributing to AD progression [62]. Thus, microglial responses to AD evolve over time, with both beneficial and detrimental consequences.

### 3.1. Microglial Effects on Aβ Pathology

Microglia play a pivotal role in Aβ uptake and degradation. Aβ can act as an “eat-me” signal, promoting its own clearance via microglial receptors. CD36, a scavenger receptor on microglia, is critical for Aβ recognition and uptake. CD36 expression is upregulated in AD brains and promotes microglial recruitment and activation [63,64]. However, in AD, impaired recycling of CD36 via the retromer complex—regulated by Beclin-1—reduces Aβ degradation despite increased surface levels [65]. Notably, deletion of CD36 reduces cerebral amyloid angiopathy in amyloid precursor protein (APP)-overexpressing mice, likely by enhancing brain-to-blood clearance via endothelial CD36 rather than microglial mechanisms [66].

LC3-associated endocytosis (LANDO) enhances Aβ receptor recycling in microglia, promoting efficient Aβ clearance. Conversely, LANDO-deficient AD mice display neurodegeneration and memory deficits [67]. Aging exacerbates Aβ pathology by increasing microglial expression of Nogo receptor (NgR), which impairs clearance; NgR-deficient AD mice show reduced amyloid burden and improved cognition [68].

The β-site APP-cleaving enzyme-1 (BACE1) catalyzes the first step of Aβ generation by cleaving APP. While its inhibition is primarily viewed as a neuronal strategy to reduce Aβ production, recent evidence demonstrates that microglial BACE1 inhibition triggers a cell-intrinsic shift toward a “disease-associated microglia-1 (DAM-1)” phenotype [69]. Mechanistically, lowering BACE1 activity in microglia attenuates basal inflammatory signaling and enhances TREM2–DAP12 and APOE-dependent transcriptional programs. This reprogramming increases lysosomal biogenesis, upregulates phagocytic receptors such as CD36, MerTK, and LPL, and boosts endosomal–lysosomal flux, collectively augmenting Aβ internalization and degradation. In AD mouse models, this DAM-1 polarization correlates with reduced plaque burden and improved synaptic and cognitive performance [69].

Microglial clearance efficacy depends not only on intrinsic activation state but also on effective migration toward Aβ deposits. Astrocytes respond to local Aβ accumulation and cytokine stress by secreting interleukin-3 (IL-3). IL-3 binds to IL-3 receptor α (IL-3Rα) expressed on neighboring microglia, activating downstream JAK2–STAT5 and PI3K–AKT pathways. This signaling cascade enhances cytoskeletal remodeling, chemotaxis, and survival, thereby facilitating directed migration of DAM-like microglia to Aβ plaques [70]. Once in proximity, IL-3 signaling synergizes with BACE1-inhibition-induced lysosomal activation to maximize local Aβ clearance. Thus, astrocyte-microglia crosstalk via IL-3 acts as a spatial recruitment signal that complements the functional reprogramming initiated within microglia by BACE1 inhibition.

Aβ degradation can also be limited by lysosomal dysfunction. In the TgCRND8 mouse model, impaired lysosomal activity led to inefficient Aβ digestion. Deletion of Cystatin B, a natural inhibitor of lysosomal proteases, reduced Aβ40 and Aβ42 accumulation and rescued cognitive deficits [71,72].

### 3.2. Effect of Microglia on Tau Pathology

In AD, tau can be secreted by neurons into the extracellular space, contributing to the progression of pathology [73]. Microglia are key mediators of tau propagation, especially from the entorhinal cortex, by engulfing tau aggregates and subsequently releasing processed tau via exosomes [74].

Blocking exosome release, either by inhibiting neutral sphingomyelinase or through microglial ablation, prevented extracellular tau transmission and its associated neurotoxicity in vivo [75]. Extracellular tau interacts with the CX3CR1 receptor on microglia; however, CX3CR1 knockout mice exhibit impaired tau clearance [76]. Notably, tau phosphorylated at serine 396 (pS396), which accumulates in AD, displays reduced binding affinity for CX3CR1 [76,77].

Microglia can further exacerbate tau pathology by promoting neuroinflammation through activation of the NLRP3 (NOD-like receptor family, pyrin domain containing 3) inflammasome [78] and NF-κB signaling pathways [79]. Deficient autophagy in AD microglia may also hinder tau degradation and promote its spread [80,81].

**Figure 2:**
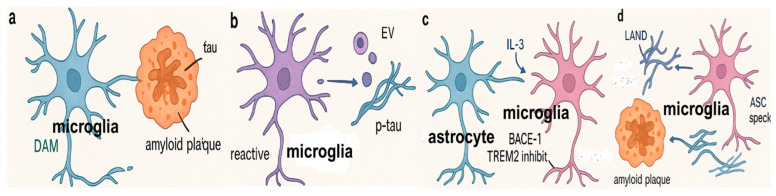
Microglial Modulation of Amyloid-β and Tau Pathology in Alzheimer’s Disease. Schematic overview of microglial functions in Alzheimer’s disease. (**a**). TREM2-dependent disease-associated microglia (DAM) encircle amyloid plaques, promoting their compaction and forming a protective barrier that restricts local tau seeding and spread. (**b**). Reactive microglia accelerate tau propagation through inflammatory signaling cascades, including NLRP3 inflammasome activation and NF-κB-driven cytokine release, which promote tau hyperphosphorylation, extracellular release, and uptake in recipient cells. Microglia in the neurodegenerative state secrete extracellular vesicles (EV) enriched in phosphorylated tau (p-tau), thereby facilitating intercellular propagation and accelerating the spread of tau pathology. (**c**). Astrocyte-derived IL-3 promotes microglial migration and Aβ clearance through IL-3Rα signaling. In parallel, TREM2 activation and BACE-1 inhibition support the transition of microglia toward protective DAM sub-phenotypes, while LC3-associated endocytosis (LANDO) sustains receptor recycling required for efficient Aβ uptake and degradation. (**d**). Activated microglia release apoptosis-associated speck-like protein containing a CARD (ASC) specks as a by-product of NLRP3 inflammasome activation. These extracellular ASC aggregates bind Aβ, acting as nucleation sites that accelerate Aβ fibrillization and promote plaque spreading, thereby creating a self-amplifying cycle of neuroinflammation and amyloid pathology. LAND (LC3-associated phagocytosis of apoptotic neurons) is a microglial process where LC3 conjugation to phagosomes enhances degradation of dead neurons, preventing secondary necrosis and limiting inflammation.

Moreover, DAM is known to hyper-secrete extracellular vesicles containing phosphorylated tau (pTau), enhancing tau propagation in AD mouse models [82]. Inhibition of tau-containing extracellular vesicle secretion by microglia ameliorated tau pathology and cognitive deficits in P301S tau transgenic mice [83] (Figure 2).

### 3.3. Molecular Sensors for Microglial Phagocytosis in Alzheimer’s Disease

In addition to surface receptors, microglia express a range of molecular sensors that regulate their phagocytic activity and responses to Aβ deposition (Table 1). Mice lacking NLRP3 or caspase-1 show enhanced Aβ clearance and protection from spatial memory deficits when crossed with familial AD models, highlighting the inflammasome’s dual role in inflammation and phagocytosis [84]. Furthermore, inflammasome-derived ASC (Apoptosis-associated Speck-like protein containing a CARD) specks released by degenerating microglia can bind to Aβ oligomers and promote plaque formation, thereby impairing microglial clearance mechanisms [85]. Scavenger receptors such as Scavenger receptor A (SR-A) and class A1 scavenger receptor (Scara1) also mediate Aβ uptake but are downregulated in aged APP transgenic mice, correlating with diminished phagocytosis and heightened inflammation [86]. Deletion of Scara1 worsens Aβ pathology [87]. The complement system supports Aβ clearance through C3 interaction with SR-A, as C3 deletion increases amyloid burden in AD models [88]. Additionally, signal-regulatory protein beta-1 (SIRPβ1), upregulated in microglia by interferons β and γ, enhances Aβ and cellular debris phagocytosis, likely via cytoskeletal remodeling [89]. Despite their clearance role, microglia may paradoxically contribute to plaque formation and spread, as sustained microglial depletion in AD mice reduces amyloid deposition [85], and ASC specks or infiltrating microglia can seed and propagate Aβ aggregates to previously unaffected brain regions [90].

## 4. Impaired Phagocytosis in Alzheimer’s Disease

### 4.1. Mechanisms Underlying Impaired Phagocytosis in Alzheimer’s Disease

In AD, the phagocytic capacity of microglia is frequently impaired, leading to the accumulation of pathological proteins and accelerating neurodegeneration. This impairment is driven by genetic mutations, dysfunction of phagocytic receptors, chronic inflammation, oxidative stress, and age-related changes in microglial function.

#### 4.1.1. Genetic Factors and Receptor Dysfunction

Several genetic risk factors for AD, including mutations in *Clusterin*, *ABCA7*, *Progranulin*, *APOE*, *TREM2*, and *CD33*, are associated with altered microglial phagocytosis. Defects in this process can lead to the aberrant clearance of stressed, yet viable neurons—contributing to neuronal loss [97,98,99].

TREM2-dependent microglial activation is essential for the defense against Aβ and tau pathology. Inhibition or loss-of-function mutations of TREM2, such as the R47H variant, impair microglial phagocytosis of amyloid plaques and increase amyloid seeding in mouse models of AD [100]. The R47H variant is associated with a 2- to 4-fold increased risk of AD [101]. Leyns et al. [102] demonstrated that both TREM2 deficiency and the TREM2 R47H variant enhance the vulnerability of dystrophic neurons near Aβ plaques to tau seeding and propagation. This is accompanied by increased Aβ42 accumulation and reduced microglial activation around plaques. Additional TREM2 variants (R62H, T66M, H157Y, D87N) also increase AD risk by reducing TREM2 expression or function [103,104].

TREM2 is proteolytically cleaved to release a soluble form (sTREM2), detectable in cerebrospinal fluid (CSF) and plasma. Elevated CSF sTREM2 correlates with slower Aβ accumulation and cognitive decline, suggesting a protective role [105]. sTREM2 enhances microglial recruitment, promotes Aβ phagocytosis, and reduces neurotoxicity [106]. However, certain mutations, such as H157Y, increase sTREM2 levels while paradoxically raising AD risk [107]. Additionally, sTREM2 can bind neurons and inhibit long-term potentiation, negatively impacting cognition [108], highlighting its complex role.

The *APOE4* allele, a major genetic risk factor for AD, impairs microglial Aβ clearance, promotes plaque formation, and disrupts lipid metabolism—further diminishing phagocytic efficiency [109,110].

#### 4.1.2. Chronic Inflammation, Oxidative Stress and Microglia Senescence

In the AD brain, prolonged microglial activation leads to persistent release of pro-inflammatory cytokines such as TNF-α, IL-1β, and IL-6. While these cytokines are important in acute immune responses, their chronic expression downregulates phagocytic receptors (e.g., TREM2, CD36) and impairs microglial ability to recognize and engulf Aβ and damaged cells [111]. Chronic inflammation also activates the NLRP3 inflammasome, which reduces phagocytic capacity and increases Aβ burden [112]. Overactivation of the complement system in AD contributes to the inappropriate phagocytosis of healthy synapses. Proteins like C1q are dysregulated, leading to chronic inflammation and impaired microglial debris clearance, exacerbating disease progression [36].

Oxidative stress—an imbalance between reactive oxygen species (ROS) production and antioxidant defenses—damages cellular components, including lipids, proteins, and DNA [113]. In microglia, oxidative stress can impair phagocytic function by oxidizing receptors like CD36 and inhibiting signaling pathways such as phosphoinositide 3-kinase (PI3K)/Protein kinase B (PKB) (Akt), essential for engulfment and motility [114]. ROS also promote Aβ and tau aggregation, increasing their neurotoxicity and further overwhelming microglial clearance mechanisms.

Both long-term inflammation and ROS promote microglial senescence, characterized by decreased responsiveness and reduced phagocytosis, exacerbating toxic accumulation in the brain. Aging shifts microglia toward a pro-inflammatory phenotype, with reduced phagocytic capability and increased cytokine production. This contributes to the chronic neuroinflammation observed in AD and reduces clearance of Aβ and cellular debris [115].

Advanced glycation end products (AGEs), which accumulate in aging and AD, bind to RAGE, triggering inflammation and oxidative stress [116]. RAGE, expressed by microglia and neurons, mediates Aβ uptake and toxicity [117,118]. However, overexpression of RAGE in microglia increases Aβ accumulation and neuroinflammation while impairing learning [94]. It remains unclear whether this is due to impaired phagocytosis or an exaggerated inflammatory response. Loss of CD47, another phagocytic co-receptor, with aging further diminishes microglial recognition of Aβ [119], though CD47 deficiency does not affect cytokine release [120].

#### 4.1.3. Dysfunction of Phagocytic Receptors and Signaling Pathways

Several phagocytic receptors and their downstream signaling cascades are disrupted in AD, resulting in impaired microglial clearance of apoptotic cells, debris, and Aβ. One of the most studied receptors, TREM2, plays a crucial role in mediating microglial activation and phagocytosis by signaling through the DAP12–PI3K/Akt and MAPK pathways, which promote cytoskeletal rearrangement and engulfment processes. The TREM2–DAP12–PI3K/Akt axis activates downstream effectors that drive actin cytoskeletal remodeling, essential for efficient phagosome formation and target engulfment. Loss-of-function mutations or downregulation of TREM2 compromise these pathways, leading to reduced microglial clustering around plaques, defective Aβ uptake, and diminished metabolic fitness [121]. Similarly, CD36, a scavenger receptor involved in recognizing oxidized lipids and Aβ fibrils, shows decreased expression and functionality in AD, further impairing microglial migration, phagocytic capacity, and lipid metabolism [36]. Dysregulation of complement receptors (e.g., CR3) and Fc receptors also contributes to aberrant clearance mechanisms, amplifying neuroinflammatory signaling and synaptic loss. Conversely, chronic inflammatory cues can shift signaling toward NF-κB activation, which acts as a molecular switch—suppressing phagocytosis while promoting pro-inflammatory cytokine release. Such pathway imbalance perpetuates neuroinflammation and metabolic stress within microglia. Furthermore, DAM exhibits metabolic reprogramming, characterized by a shift from oxidative phosphorylation to glycolysis during inflammation and impaired mitochondrial respiration in aging brains, both of which reduce energy availability for sustained phagocytic activity [122]. Collectively, the dysfunction of these receptor-mediated and intracellular signaling pathways disrupts the delicate balance between microglial clearance and inflammation, thereby exacerbating Aβ accumulation and neurodegeneration in AD.

### 4.2. Consequences of Impaired Phagocytosis in Alzheimer’s Disease

#### 4.2.1. Accumulation of Amyloid-Beta Plaques and Neurofibrillary Tangles

Under normal conditions, microglia clear Aβ via phagocytosis and lysosomal degradation. Impaired microglial function in AD results in Aβ accumulation, extracellular plaque formation, and subsequent neuroinflammation. These plaques also induce tau hyperphosphorylation and neurofibrillary tangle formation, intensifying neuronal injury [111,114]. Deficient phagocytic activity allows tau aggregates to persist and propagate, contributing to neuronal dysfunction, synaptic loss, and cell death. The trans-synaptic spread of tau pathology further facilitates the progression of neurodegeneration [111]. Failure to clear apoptotic cells and debris leads to sustained inflammation through the release of DAMPs [20]. This creates a feedback loop wherein chronic inflammation impairs phagocytosis, exacerbating toxic accumulation and neuronal damage.

#### 4.2.2. Microglial Dysfunction Impairs Neuronal Plasticity

In AD, microglia contribute to neuronal dysfunction not only through the release of pro-inflammatory mediators but also by remodeling and degrading the extracellular matrix, particularly perineuronal nets (PNNs) [123,124]. PNNs are lattice-like structures composed of chondroitin sulfate proteoglycans (CSPGs), hyaluronan, tenascin-R, and link proteins that enwrap specific populations of neurons, especially parvalbumin-positive interneurons. These structures emerge during postnatal development to stabilize synaptic contacts, limit excessive plasticity, and maintain excitation–inhibition balance within neuronal circuits [124].

**Figure 3:**
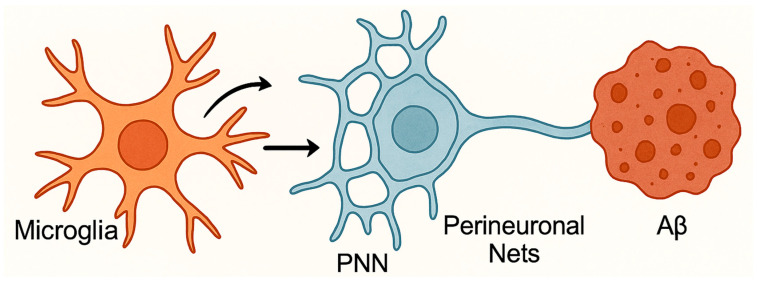
Microglial degradation of perineuronal nets in Alzheimer’s disease. In Alzheimer’s disease, chronic activation of microglia leads to excessive release of MMP-2, MMP-9, and cathepsins, which degrade perineuronal nets (PNNs), key extracellular matrix structures that regulate synaptic plasticity. PNN loss correlates with Aβ plaque burden in both mouse models and human tissue. Microglial depletion prior to plaque formation reduces PNN loss, underscoring the role of microglia in plaque-related neuronal damage. Dysfunctional microglia thereby impair synaptic integrity and cognitive function.

Under physiological conditions, microglia contribute to ECM maintenance through regulated phagocytosis and secretion of proteases such as matrix metalloproteinases (MMPs) [125]. However, in the context of AD, chronic activation of microglia driven by amyloid-β deposition, tau pathology, and persistent inflammatory cues (e.g., IL-1β, TNF-α) leads to excessive release of MMP-2, MMP-9, and cathepsins, which enzymatically degrade PNN components [126]. This degradation destabilizes inhibitory synapses, increases neuronal excitability, and disrupts oscillatory network synchrony essential for cognitive processing (Figure 3).

### 4.3. Conflicting Evidence on Microglial Phagocytosis in Alzheimer’s Disease

Microglial phagocytic activity in AD follows a biphasic, context-dependent trajectory [18,127]. In the early or acute phase, exposure to Aβ aggregates, interferons, or inflammasome activation (e.g., NLRP3) triggers a reactive clearance phenotype—microglia upregulate receptors such as TREM2, CD36, and MerTK, increase lysosomal enzyme expression, and actively engulf Aβ fibrils and damaged synapses [127]. This transient enhancement serves as a compensatory mechanism to contain pathology and maintain tissue homeostasis.

As disease progresses, however, sustained stimulation and metabolic stress drive a shift toward chronic activation and phagocytic exhaustion. Microglia accumulate lipid droplets and undegraded debris, exhibit lysosomal dysfunction and mitochondrial impairment, and secrete pro-inflammatory cytokines that perpetuate neuronal injury [128]. This stage is characterized by reduced receptor expression, impaired autophagy, and the emergence of “senescent” or “dystrophic” microglia incapable of effective clearance.

Therapeutically, these two states represent distinct intervention windows: early-phase reprogramming strategies aim to preserve or enhance protective phagocytosis (e.g., via TREM2 agonists or metabolic support), whereas late-stage approaches must focus on restoring lysosomal competence, resolving inflammation, and preventing further neuronal damage [129,130,131]. recognizing this dynamic spectrum, from reactive clearance to phagocytic paralysis, is critical for developing time-sensitive, precision therapies that modulate microglial function in AD.

## 5. Targeting Microglial Phagocytosis for Treating Alzheimer’s Disease

Impaired microglial phagocytosis in AD poses a significant obstacle to developing effective therapies. Enhancing microglial phagocytosis by targeting key receptors and signaling pathways involved in this process represents a promising therapeutic strategy.

### 5.1. Targeting “Eat Me” Signals

“Eat me” signals are crucial for the clearance of apoptotic cells and maintaining brain homeostasis, making them attractive therapeutic targets in AD (Table 2). Enhancing microglia’s ability to recognize and respond to these signals could improve clearance of Aβ plaques and other toxic materials. This may involve boosting the expression or function of receptors such as TREM2, MerTK, and Axl, which recognize PS and other “eat me” signals [121,129,130,131].

However, several challenges remain. The complexity of microglial responses in AD requires careful targeting to avoid exacerbating neurodegeneration by increasing pro-inflammatory cytokine release. Therapeutic timing is critical; early intervention may be more effective than treatment at advanced stages. Additionally, long-term effects of modulating “eat me” signals must be carefully studied to avoid unintended damage to healthy neurons or disruption of brain function.

### 5.2. Targeting TREM2

Activation of TREM2 signaling enhances microglial clearance of Aβ, damaged neurons, and cellular debris, promoting tissue homeostasis and functional recovery in AD. sc RNA—seq has revealed that TREM2 drives the DAM phenotype, characterized by heightened phagocytic activity and upregulation of lipid metabolism genes [10]. In parallel, TREM2 activation supports microglial survival and migration, thereby limiting neuroinflammation through efficient debris clearance [36]. Therapeutic strategies such as TREM2 agonist antibodies, gene delivery, or expression enhancement have been shown to improve Aβ clearance and cognitive outcomes in AD mouse models [142,143,144]. For individuals carrying TREM2 mutations that impair phagocytosis, gene therapy may restore microglial function and slow disease progression [36]. However, TREM2′s role in tau pathology appears context-dependent—it may suppress tau seeding during early disease stages but promote tau propagation later [145]. Moreover, high-affinity TREM2–ApoE binding enhances microglial uptake of ApoE-bound apoptotic neurons, establishing a mechanistic link between two major genetic risk factors for AD and suggesting that TREM2-mediated debris clearance may mitigate secondary neuronal injury [146].

### 5.3. Restoring Lysosomal Function

Age-related lysosomal dysfunction in microglia impairs degradation of engulfed debris, reducing phagocytic efficiency and contributing to toxic accumulation in AD [115,147]. Enhancing lysosomal biogenesis and function through activation of transcription factor EB (TFEB) or targeting lysosomal enzymes like cathepsin B shows therapeutic potential, though clinical efficacy remains to be established [148,149].

### 5.4. Toll-like Receptor Agonists

Stimulation of microglia via TLR agonists can impair their ability to distinguish apoptotic from viable neurons, leading to increased ROS and transient PS exposure on neurons. This process promotes phagocytosis of stressed but viable neurons via increased secretion of MFG-E8 and activation of the vitronectin receptor, resulting in neuronal loss [44].

### 5.5. Targeting Oxidized Lipoproteins and Complement Pathways

Modulating interactions between oxLDL and microglial receptors, especially CD36, and regulating complement activation could reduce neuroinflammation and promote apoptotic cell clearance. Targeting these pathways has demonstrated efficacy in preclinical AD models [134].

## 6. Drug Therapies That Modulate Microglial Phagocytosis for Treating Alzheimer’s Disease

A range of therapeutic strategies have been investigated to restore or enhance microglial phagocytic function in AD, targeting both receptor-mediated signaling and intracellular pathways. Table 3 summarizes the principal approaches currently under study, including receptor agonists, modulators of intracellular cascades, and agents that reprogram microglial metabolism toward a neuroprotective phenotype.

### 6.1. TREM2-Targeting Antibodies

Several pharmaceutical companies have been developing TREM2 agonist antibodies for clinical use. AL002, a monoclonal antibody, specifically targets and activates TREM2 to enhance microglial responses in AD [143]. AL002 enhanced microglial activation and promoted the clearance of Aβ plaques, leading to a reduction in plaque load and improved cognitive function [143]. However, this antibody treatment also increased microglial clustering around amyloid plaques, a sign of enhanced microglial reactivity and activity in plaque clearance [36]. This antibody is currently undergoing clinical trials to assess its efficacy in AD patients. In early-stage trials, AL002 established an ability to enhance microglial phagocytosis of Aβ, supporting its potential as a therapeutic agent for AD [150]. In Phase 2 trials, AL002 failed to show efficacy in reducing cognitive decline and pathological hallmarks in AD patients, leading to discontinuation of its extension study [151]. The clinical development of AL002 reflects a broader trend toward immunotherapy-based approaches in AD, which aim to restore microglial function and target neuroinflammation alongside classical approaches targeting amyloid-β or tau [143].

On the other hand, TREM2 binds to ligands such as lipids and ApoE, which trigger microglial activation, migration, and phagocytosis. TREM2 inhibition may be useful in conditions where microglial phagocytosis aggravates neuronal damage and neuroinflammation, such as in the later stages of AD, when excessive microglial activation contributes to synaptic loss and cognitive decline [152]. TREM2-inhibiting antibodies have been developed to inhibit microglia activation and reduce their phagocytic activity in neurodegenerative diseases. Research study has shown that treatment with a TREM2-inhibiting antibody in AD mouse models reduced microglial activation and phagocytosis of Aβ plaques. This results in decreased neuroinflammation and synapse loss, indicating that TREM2 inhibition may have therapeutic potential in modulating microglial activity in AD [152].

### 6.2. PPARγ Agonists

Peroxisome proliferator-activated receptor gamma (PPARγ) is a nuclear receptor that regulates gene expression related to lipid metabolism, inflammation, and immune responses in microglia. PPARγ agonists exert a dual effect by stimulating anti-inflammatory responses and enhancing the microglial clearance of toxic aggregates. Activation of PPARγ reduces pro-inflammatory cytokines expression (e.g., IL-1β, TNF-α) and upregulates genes involved in phagocytosis and lipid metabolism, which are vital for microglial function. This enhanced phagocytosis facilitates the removal of Aβ, tau, and other pathogenic proteins, and at the same time reducing the chronic inflammation associated with neurodegenerative diseases [153]. Research has established that pioglitazone treatment in AD mouse models led to increased microglial-mediated clearance of Aβ, resulting in reduced plaque burden and improved spatial memory by enhancing microglial activity [153]. Pioglitazone has been evaluated in several clinical trials for its potential in treating mild cognitive impairment (MCI) and AD. The “TOMMORROW” trial is a Phase 3 study that aimed to assess whether pioglitazone could delay the onset of AD in individuals with MCI. Though the trial was eventually discontinued due to recruitment and other logistical issues, pioglitazone remains an active candidate for enhancing microglial phagocytosis in neurodegenerative diseases [154].

### 6.3. CD33 Inhibitors

CD33 is a sialic acid-binding immunoglobulin-like lectin (Siglec) expressed on microglia. It acts as a negative regulator of microglial phagocytosis, particularly in AD, where elevated CD33 expression is associated with reduced Aβ clearance and increased plaque accumulation. Inhibiting CD33 is therefore considered a promising approach to enhance microglial-mediated phagocytosis of Aβ and other aggregates [155]. Therapeutic strategies intended at inhibiting CD33 relieve this suppression, allowing microglia to effectively clear toxic proteins and cellular debris. Genetic studies revealed that individuals with reduced CD33 expression have a lower risk of developing AD, further supporting the potential of CD33 inhibitors as therapeutic agents [155]. In preclinical studies, AD mouse models with CD33 knocked out exhibited enhanced microglial phagocytosis of Aβ plaques, resulting in reduced amyloid burden and improved cognitive performance. This implies that targeting CD33 could effectively enhance microglial clearance of pathogenic proteins and slow disease progression [155,156].

### 6.4. NLRP3 Inflammasome Inhibitors

Excessive activation of the NLRP3 inflammasome drives chronic neuroinflammation, which impairs microglial function and reduces their phagocytic ability of Aβ and other pathogenic proteins. By inhibiting the NLRP3 inflammasome, microglia can shift from a pro-inflammatory, dysfunctional state to a more phagocytic phenotype, enhancing their ability to clear toxic proteins and debris [84]. MCC950, a selective NLRP3 inflammasome inhibitor, has been shown to enhance microglial phagocytosis and reduce Aβ and tau pathology in preclinical models of AD. Research studies revealed that treatment with MCC950 reduced neuroinflammation and enhanced the clearance of Aβ plaques by microglia, resulting in improved cognitive function in AD mouse models [157]. Even though MCC950 has not yet progressed to clinical trials for AD, ongoing research is focused on developing safer and more effective NLRP3 inhibitors that can be used in humans.

### 6.5. Interleukin-33

IL-33 is a cytokine recently identified as a potent enhancer of microglial phagocytosis and a promising therapeutic target for neurodegenerative diseases such as AD [158]. Expressed in the CNS, IL-33 functions as an endogenous alarmin released in response to tissue damage or stress. It activates microglia by binding to its receptor, Suppression of Tumorigenicity 2 (ST2), on the microglial surface, thereby enhancing microglial phagocytic activity, reducing neuroinflammation, and facilitating the clearance of Aβ plaques and other toxic aggregates. Experimental studies in AD mouse models have demonstrated that IL-33 administration markedly reduces Aβ plaque burden, improves cognitive performance, and promotes synaptic plasticity and neuronal survival [159]. These effects are primarily attributed to increased microglial phagocytosis and attenuated neuroinflammation, underscoring IL-33′s potential as a neuroprotective and disease-modifying agent in AD.

### 6.6. CSF1R Inhibitors

Colony-stimulating factor 1 receptor (CSF1R) is a receptor tyrosine kinase that binds to ligands such as CSF1 and IL-34, which stimulate microglial survival, proliferation, and phagocytic activity. Inhibition of CSF1R signaling results in the suppression of microglial activation and a decrease in their phagocytic activity, hence reducing neuroinflammation and neurodegeneration. PLX5622 is a selective CSF1R inhibitor, could result in a significant reduction in microglial numbers and phagocytic activity in a mouse model of AD, which was related with decreased synapse loss and neuronal death. In early-phase clinical trials, PLX5622 has shown an ability to reduce neuroinflammation and improve neurological outcomes in patients with AD and ALS. However, long-term safety and efficacy data are still being evaluated, and further research is needed to determine its full therapeutic potential in humans [160].

### 6.7. Complement System Inhibitors

Inhibiting the complement system can thus reduce microglial-mediated synaptic loss and neuroinflammation, making it a target for therapeutic intervention in neurodegenerative diseases [161]. ANX005 is a monoclonal antibody that targets C1q, the initiating protein of the classical complement pathway [162]. ANX005 reduced microglial-mediated synapse loss and improved cognitive outcomes in AD models [162]. Preliminary results in clinical trials indicate that it can safely reduce complement activation and microglial phagocytosis, with potential neuroprotective effects [163].

## 7. Barriers to Clinical Translation

Despite remarkable preclinical advances in understanding microglial biology, translating microglia-targeted therapies into clinical benefit has proven challenging. The recent clinical failure of AL002, a humanized monoclonal antibody designed to activate TREM2, exemplifies the persistent translational gap [151]. Although AL002 produced robust microglial activation, plaque compaction, and cognitive improvement in amyloid mouse models, these effects did not translate to measurable clinical efficacy in humans [150]. This disparity underscores several critical barriers: species-specific microglial responses, inadequate CNS penetration, suboptimal therapeutic timing, and pathological heterogeneity across AD subtypes [151]. Similarly, pioglitazone, a PPAR-γ agonist initially shown to suppress microglial inflammation and improve cognition in transgenic mice, failed to demonstrate significant benefit in human clinical trials [154]. The lack of efficacy likely reflects poor blood brain barriers permeability, the late disease stage of recruited participants, and systemic metabolic side effects that limited dosage escalation [154]. Collectively, these examples illustrate the need to move beyond linear translation from murine models to human trials and instead adopt integrative, human-relevant experimental systems that reflect the temporal, cellular, and molecular diversity of human AD.

### 7.1. Timing and Disease Stage

Therapeutic timing is one of the most decisive—and often overlooked—determinants of microglial intervention outcomes [127]. Early-stage modulation of microglia, when pathology is largely confined to amyloid deposition and synaptic architecture is preserved, can enhance Aβ clearance, limit inflammation, and maintain neuroprotective functions [127]. In contrast, late-stage activation, once neurodegeneration and synaptic loss are established, may exacerbate excitotoxicity, propagate neuroinflammation, and worsen clinical decline [128]. This stage-dependent dichotomy implies that temporal stratification is essential for clinical success. Biomarkers such as sTREM2, which reflect microglial activation, or TSPO-PET imaging, which visualizes neuroinflammation in vivo, could help dynamically classify patients and determine optimal therapeutic windows [130]. Combining such biomarkers with plasma proteomic or lipidomic signatures may support precision dosing strategies, enabling microglial therapies to be delivered when they are most likely to promote clearance rather than destruction [130].

### 7.2. Species Gaps and Model Limitations

Most preclinical data derive from young, amyloid-only mouse models that lack the chronic, multifactorial environment of human AD. These models typically display uniform genetic backgrounds, limited comorbidity, and rapid pathology, conditions that rarely mirror human disease [164]. Recent snRNA-seq and PET imaging studies in human AD have revealed activation profiles that diverge from murine DAM signatures [165,166]. Moreover, human iPSC-derived microglia display distinct lipid metabolism, cytokine responsiveness, and phagocytic regulation compared with murine counterparts, reflecting evolutionary divergence in immune signaling and energy homeostasis [167]. These findings highlight the necessity of integrating human-derived in vitro systems—such as iPSC microglia co-cultured with neurons and astrocytes—into translational pipelines [168]. Such models can bridge preclinical–clinical gaps by recapitulating patient-specific genetics, age-related epigenetic signatures, and metabolic constraints absent in rodents.

### 7.3. Heterogeneity Across AD Subtypes

**Figure 4:**
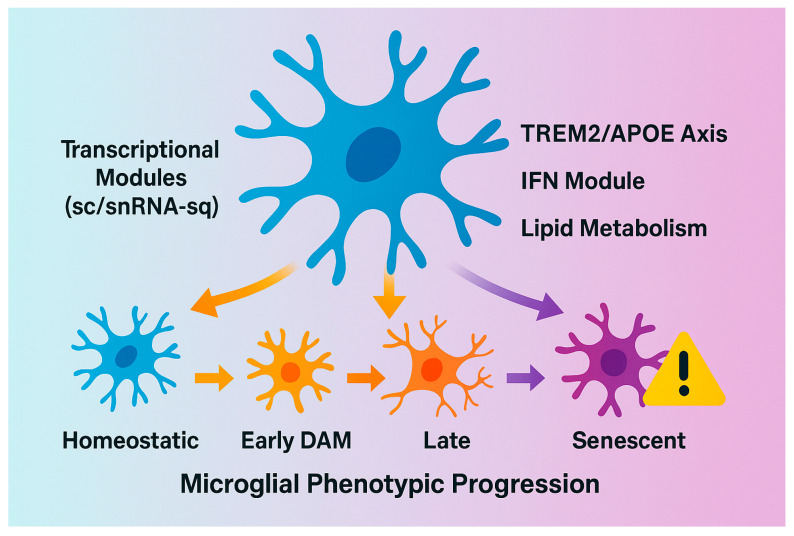
Schematic overview of microglial phenotypic transitions and associated transcriptional modules in Alzheimer’s disease. This figure illustrates the progressive transformation of microglia from a homeostatic state to successive disease-associated phenotypes observed during AD progression, as revealed by single-cell and single-nucleus RNA sequencing (sc/snRNA-seq) studies. Under physiological conditions, homeostatic microglia maintain synaptic integrity, perform debris clearance, and express canonical markers such as P2RY12, TMEM119, and CX3CR1. In the early stages of AD, microglia adopt an early Disease-Associated Microglia (DAM) phenotype characterized by the activation of the TREM2–APOE axis, upregulation of Apoe, Tyrobp, Lpl, and Trem2, and engagement of lipid metabolism and phagocytic pathways to clear amyloid deposits. As pathology advances, these cells transition into late neurodegenerative microglia, defined by increased expression of pro-inflammatory and interferon (IFN)-responsive genes (Ifitm3, Irf7, Isg15) and a decline in phagocytic efficiency. Ultimately, chronic activation and metabolic exhaustion lead to the emergence of senescent microglia, exhibiting reduced motility, impaired mitochondrial function, and elevated expression of senescence and stress markers such as Cdkn1a (p21) and SA-β-gal activity.

AD is not a singular entity, but a spectrum of overlapping subtypes distinguished by genetic, metabolic, and inflammatory signatures. Integrating omics datasets suggests a continuum rather than discrete subtypes, with transitions modulated by metabolic and inflammatory cues [169,170]. We propose a conceptual framework linking early ‘protective DAM’ to late ‘phagocytic exhaustion’ states, reconciling apparent discrepancies across datasets (Figure 4).

APOE, highly expressed by astrocytes and reactive microglia, serves as a lipid transporter and co-factor for TREM2-mediated phagocytosis. Different APOE isoforms confer distinct microglial phenotypes: APOE3 promotes efficient lipid handling and stabilizes TREM2–APOE interactions, thereby maintaining the DAM-1 transcriptional signature and supporting lysosomal integrity. In contrast, APOE4 destabilizes lipid metabolism, induces endoplasmic-reticulum stress, and impairs autophagic flux, leading to reduced Aβ uptake and incomplete degradation [171]. Consequently, APOE3-expressing microglia sustain higher phagocytic capacity and stronger anti-inflammatory feedback loops, whereas APOE4 biases microglia toward a chronically inflamed, less efficient state. APOE4 carriers exhibit heightened baseline microglial reactivity and impaired lipid clearance, predisposing them to exaggerated neuroinflammatory responses following therapeutic stimulation. Similarly, mixed pathologies—such as coexisting vascular injury, α-synuclein accumulation, or TDP-43 pathology—may alter microglial phenotypes and obscure treatment effects in unstratified cohorts. Failure to account for such heterogeneity may explain inconsistent clinical outcomes, as microglial-targeting agents could exert beneficial effects in one pathological context and detrimental ones in another. Precision stratification using genetic risk profiling (e.g., APOE, TREM2 variants), fluid biomarkers, and neuroimaging markers will be critical to identify the subgroups most likely to respond to microglial interventions [130].

### 7.4. Off-Target Toxicity and Overactivation

A further translational barrier lies in the narrow therapeutic window between beneficial microglial activation and overactivation-associated neurotoxicity. Excessive stimulation of immune receptors such as TREM2, CD33, or TLR4 can drive cytokine release, synaptic stripping, and astrocyte reactivity, culminating in neuronal loss [172]. This risk is compounded by limited CNS penetrance of large-molecule therapeutics, which may lead to uneven regional exposure and unpredictable microglial activation patterns. Balancing these competing effects will require precise dosing regimens and biomarker-guided monitoring to avoid tipping microglia from restorative to destructive states [130].

### 7.5. Emerging Metabolic and Peripheral Influences

Recent studies emphasize the influence of microglial metabolism—particularly lipid droplet accumulation, mitochondrial dysfunction, and oxidative stress—on phagocytic efficacy and inflammatory tone. Lipid-laden microglia accumulate in aged and AD brains, where defective β-oxidation impairs energy production and perpetuates chronic inflammation [173,174]. Pharmacological interventions that restore metabolic flexibility, such as AMPK activators or mitochondrial biogenesis enhancers, could reinvigorate microglial clearance functions. Moreover, systemic factors are increasingly recognized as modulators of microglial behavior. Gut-derived metabolites (e.g., short-chain fatty acids, tryptophan derivatives) and peripheral immune training can reprogram microglial transcriptional states through circulating cytokines and microbial by-products [175,,176]. This emerging neuroimmune axis offers new therapeutic entry points—potentially enabling indirect modulation of microglia via peripheral or dietary interventions, thereby circumventing the challenge of CNS drug delivery.

## 8. Conclusions

Phagocytosis is a critical function of microglia that supports brain development by regulating neuronal populations and synaptic connections, while also maintaining homeostasis through the clearance of invading pathogens and age-associated protein aggregates. Impaired microglial phagocytosis contributes to AD progression by facilitating toxic protein accumulation, sustaining chronic neuroinflammation, and driving neuronal dysfunction. Despite advances in understanding microglial biology, key aspects—such as heterogeneity and phenotypic transitions across disease stages—remain incompletely defined. Although many studies report reduced phagocytosis of aggregated proteins and apoptotic cells in AD, some evidence suggests phagocytic activity may actually increase under inflammatory conditions, potentially amplifying neurodegeneration. These complexities underscore major translational barriers: effective microglial therapeutics require frameworks that integrate disease stage, human-relevant models, metabolic context, and patient variability. Overcoming these challenges will rely on longitudinal, biomarker-informed clinical trials, cross-species transcriptomic validation, and pharmacokinetic designs aligned with microglial functional states. Emerging work using human-derived microglia—particularly those generated from induced pluripotent stem cells (iPSCs), alongside refined postmortem isolation techniques, is providing unprecedented insight into human-specific microglial mechanisms in neurodegeneration. Future research should focus on identifying molecular regulators that define protective versus pathogenic microglial phenotypes and on developing interventions that reprogram microglia toward a neuroprotective state early in disease. A more comprehensive understanding of microglial phagocytosis in AD could ultimately enable the design of precision therapies capable of halting or even reversing disease progression.

## Figures and Tables

**Table 1:** Overview of Molecular Sensors Involved in the Regulation of Microglial Phagocytosis in Alzheimer’s Disease.

Molecule	Substrate	Effect on Phagocytosis	Reference(s)
CXCL1/CX3CR1	Tau	Upregulation	[77]
TREM2	Aβ	Upregulation	[36,91]
	Apoptotic cells	Upregulation	
MFG-E8	Apoptotic cells	Upregulation	[41,42,92]
C3	Aβ	Upregulation	[88]
SIRPβ1	Aβ	Upregulation	[93]
Scara1	Aβ	Upregulation	[87]
RAGE	Aβ	Upregulation	[94,95]
	Aβ	Downregulation	
CD47	Aβ	Upregulation	[52]
	Apoptotic cells	Downregulation	
NLRP3	Aβ	Downregulation	[84]
Caspase-1	Aβ	Downregulation	[84]
ASC	Aβ	Downregulation	[85]
Cystatin B	Aβ	Downregulation	[96]
P2Y6	Aβ	Upregulation	[40]

**Table 2:** Key “Eat me” Signals in Alzheimer’s Disease Brain.

**Phosphatidylserine (PS)**	Enhancing PS-receptor interaction may restore phagocytic function [132].
**Calreticulin**	Exposed on stressed neurons due to Aβ and tau toxicity; insufficient clearance contributes to neuroinflammation [133]. Enhancing microglial sensitivity to calreticulin may reduce debris accumulation.
**Oxidized LDL (oxLDL)**	Oxidative stress increases oxLDL formation, which binds microglial receptors like CD36. Boosting CD36 function may improve clearance and reduce inflammation [134].
**Complement Proteins**	Dysregulated complement activation contributes to neuroinflammation. Therapeutic inhibition of excessive complement activity while preserving clearance functions shows promise [134].
**Amyloid-beta (Aβ)**	Immunotherapies designed to promote Aβ clearance are now in clinical use. This involves monoclonal antibodies, e.g., Aducanumab (Aduhelm), Lecanemab (Leqembi), Donanemab, facilitating their clearance through microglial phagocytosis or peripheral sink mechanisms [135].
**Neuron-Specific Enolase (NSE)**	Elevated NSE reflects neuronal damage and overwhelmed microglial clearance; enhancing response to NSE could mitigate inflammation [136].
**High Mobility Group Box 1 (HMGB1)**	Targeting HMGB1 or its receptors (e.g., RAGE, TLR4) may modulate inflammation while supporting clearance [137,138].
**Signal Regulatory Protein Alpha (SIRPα)**	Blocking CD47-SIRPα interaction enhances microglial clearance and reduces inflammation, representing a novel therapeutic target [139,140,141].

**Table 3:** Summary of Therapeutic Strategies Targeting Microglial Phagocytosis in AD.

Strategy	Mechanism	Outcome in Preclinical/Clinical Models
TREM2 Agonists (e.g., AL002)	Enhances phagocytosis, promotes DAM phenotype	Reduced Aβ, improved cognition
PPARγ Agonists (e.g., Pioglitazone)	Anti-inflammatory and pro-phagocytic gene regulation	Decreased plaque burden, improved memory
CD33 Inhibitors	Blocks negative regulation of phagocytosis	Enhanced Aβ clearance, cognitive improvement
NLRP3 Inhibitors (e.g., MCC950)	Reduces inflammasome activation	Reduced inflammation, enhanced Aβ/tau clearance
IL-33	Enhances phagocytosis via ST2 receptor	Reduced Aβ, improved synaptic health
CSF1R Inhibitors (e.g., PLX5622)	Depletes or resets microglial populations	Reduced neuroinflammation, potential repopulation benefit

## Data Availability

Not Applicable.
